# Improved estimates for the triangle inequality

**DOI:** 10.1186/s13660-016-1281-z

**Published:** 2017-01-13

**Authors:** Nicuşor Minculete, Radu Păltănea

**Affiliations:** Faculty of Mathematics and Computer Science, Transilvania University of Braşov, Str. Iuliu Maniu, nr. 50, Braşov, 500091 Romania

**Keywords:** 46B99, 26D15, 46C50, 46C05, norm inequalities, triangle inequality, Tapia semi-product, inner product spaces, norm-angular distance

## Abstract

We obtain refined estimates of the triangle inequality in a normed space using integrals and the Tapia semi-product. The particular case of an inner product space is discussed in more detail.

## Introduction

The theory of inequalities plays an important role in many areas of Mathematics. Among the most used inequalities we find the triangle inequality. This inequality is the following:

$$\Vert x+y\Vert \le \Vert x\Vert +\Vert y\Vert , $$

for any vectors *x* and *y* in the normed linear space $(X,\Vert \cdot \Vert )$ over the real numbers or complex numbers. Its continuous version is,

$$\biggl\Vert \int_{a}^{b} f(t)\,dt\biggr\Vert \le \int_{a}^{b}\bigl\Vert f(t)\bigr\Vert \,dt, $$

where $f: [a, b]\subset\mathbb{R}\to X$ is a strongly measurable function on the compact interval $[a, b]$ with values in a Banach space *X* and $\Vert f(\cdot)\Vert $ is the Lebesgue integrable on $[a, b]$.

Diaz and Metcalf [1] proved a reverse of the triangle inequality in the particular case of spaces with inner product. Several other reverses of the triangle inequality were obtained by Dragomir in [2]. Also, in [2] some inequalities for the continuous version of the triangle inequality using the Bochner integrable functions are given. In [3], Rajić gives a characterization of the norm triangle equality in pre-Hilbert $C^{\star }$-modules. In [4, 5], Maligranda proved a refinement of the triangle inequality as follows.

### Theorem A

*For nonzero vectors* *x* *and* *y* *in a normed space* $(X,\Vert \cdot \Vert )$ *it is true that*

1 $$\begin{aligned} \biggl(2-\biggl\Vert \frac{x}{\Vert x\Vert }+\frac {y}{\Vert y\Vert }\biggr\Vert \biggr)\min\bigl\{ \Vert x\Vert ,\Vert y\Vert \bigr\} \le&\Vert x\Vert + \Vert y\Vert -\Vert x+y\Vert \\ \le& \biggl(2-\biggl\Vert \frac {x}{\Vert x\Vert }+ \frac{y}{\Vert y\Vert }\biggr\Vert \biggr)\max\bigl\{ \Vert x\Vert ,\Vert y \Vert \bigr\} . \end{aligned}$$

In [6] Kato, Saito and Tamura proved the sharp triangle inequality and reverse inequality in Banach space for nonzero elements $x_{1},x_{2},\ldots,x_{n}\in X$, which is in fact a generalization of Maligranda’s inequality. Another extension of Maligranda’s inequality for *n* elements in a Banach space was obtained in Mitani and Saito [7]. The problem of characterization of all intermediate values *C* satisfying $0\le C\le\sum_{k=1}^{n}\Vert x_{k}\Vert -\Vert \sum_{k=1}^{n} x_{k}\Vert $, for $x_{1},x_{2},\ldots ,x_{n}$ in a Banach space is studied by Mineno, Nakamura and Ohwada [8], Dadipour *et al.* [9], Sano *et al.* [10] and others. For other different results about the triangle inequality we mention only [11–15].

The main aim of the present paper is to provide an improvement of the inequality due to Maligranda. Some other estimates which follow from the triangle inequality are also presented. Moreover, we can rewrite them as estimates for the so-called *norm-angular distance* or *Clarkson distance* (see *e.g.* [4]) between nonzero *x* and *y* as $\alpha[x,y]=\Vert \frac{x}{\Vert x\Vert }-\frac{y}{\Vert y\Vert }\Vert $. This distance was generalized to the *p*-angular distance in normed space in [4]. In [16], Dragomir characterizes this distance obtaining new bounds for it. A survey on the results for bounds for the angular distance, so named Dunkl-Williams type theorems (see [17]), is given by Moslehian *et al.* [18].

## Estimates of the triangle inequality using integrals

Let $(X,\Vert \cdot \Vert )$ be a real normed space. The following lemma is evident.

### Lemma 1

*For any* $x,y\in X$, *the function* $g(s)=\Vert x+sy\Vert $, $s\in\mathbb{R,}$ *is convex*.

### Theorem 1

*For any* $x,y\in X$ *we have*

(i) $\Vert x+y\Vert \le2\int_{0}^{1}\Vert (1-\lambda)x+\lambda y\Vert \,d\lambda\le \Vert x\Vert +\Vert y\Vert $,
(ii) $\Vert x\Vert +\Vert y\Vert +\Vert x+y\Vert \ge4\int_{0}^{1}\Vert (1-\lambda )x+\lambda y\Vert \,d\lambda$.

### Proof

(i) Let $a,b\in\mathbb{R}$, $a< b$ arbitrarily chosen. Since the function $g(s)=\Vert z+su\Vert $, $s\in\mathbb{R}$ is convex, for any fixed $u,z\in X$ we apply the Hermite-Hadamard inequality, see *e.g.* [19, 20] for $a< b$:

$$g \biggl(\frac{a+b}{2} \biggr)\le\frac{1}{b-a} \int_{a}^{b}g(s)\,ds\le\frac {g(a)+g(b)}{2}. $$

Therefore we obtain

2 $$ \biggl\Vert z+\frac{a+b}{2}u\biggr\Vert \le \frac{1}{b-a} \int_{a}^{b}\Vert z+su\Vert \,ds\le \frac{\Vert z+au\Vert +\Vert z+b u\Vert }{2}. $$

For any $a< b$ and any $x,y\in X$, $u=\frac{1}{a-b}(x-y)$ and $z=\frac{1}{a-b}(ay-bx)$ such that $x=z+au$ and $y=z+bu$. By replacing in inequality (2) we deduce the inequality

$$\frac{1}{2}\Vert x+y\Vert \le\frac{1}{(b-a)^{2}} \int_{a}^{b}\bigl\Vert bx-ay+s(y-x)\bigr\Vert \,ds \le\frac{\Vert x\Vert +\Vert y\Vert }{2}. $$

If we multiply these inequalities by 2 and make the change of variable $s=(1-\lambda)a+\lambda b$ in the integral, we arrive at relation (i).

(ii) If we apply the Hammer-Bullen inequality, see *e.g.* [19], for $a< b$ we obtain

$$\frac{g(a)+g(b)}{2}+g \biggl(\frac{a+b}{2} \biggr)\ge\frac{2}{b-a} \int_{a}^{b}g(s)\,ds. $$

Now, if we proceed similarly to (i) we obtain relation (ii). □

### Example 1

Let $X=\mathbb{R}^{2}$, endowed with the norm $\Vert (x_{1},x_{2})\Vert =\max\{\vert x_{1}\vert ,\vert x_{2}\vert \}$. Let $\eta,\mu\in[0,1]$ and define $x=(1,1)$, $y=(-\mu,\eta\mu)$. We have $\Vert x\Vert =1$, $\Vert y\Vert =\mu$, $\Vert x+y\Vert =1+\eta\mu$. For $\lambda\in[0,1]$, since $(1-\lambda)(-\lambda\eta)\le0$ and $(1-\lambda)\lambda\eta\mu \ge0$, we obtain $\Vert (1-\lambda)x+\lambda y\Vert =\Vert (1-\lambda-\lambda\mu,1-\lambda+\lambda\eta\mu)\Vert =1-\lambda+\lambda\eta\mu$ and then

$$2 \int_{0}^{1}\bigl\Vert (1-\lambda)x+\lambda y\bigr\Vert \,d\lambda =2 \int_{0}^{1}(1-\lambda+\lambda\eta\mu)\,d\lambda=1+ \eta\mu. $$

Hence, relations (i) and (ii) become

$$\mathrm{(i)} \quad 1+\eta\mu\le1+\eta\mu\le1+\mu, \quad \quad \mathrm{(ii)}\quad 2+\mu+\eta\mu\ge 2+2\eta\mu. $$

### Remark 1

Since the parameters *η*, *μ* can be taken arbitrarily in the interval $[0,1]$ in Example 1, it follows that the constants in front of the terms of inequalities (i) and (ii) in Theorem 1 are optimal.

### Corollary 1

*For nonzero elements* *x*, *y* *from a space with inner product* $X=(X,\langle\cdot,\cdot\rangle)$ *and any* $a,b\in\mathbb {R}$, $a< b$, *we have*

3 $$\begin{aligned} \frac{2(\Vert x\Vert \Vert y\Vert -\langle x,y\rangle)}{\Vert x\Vert +\Vert y\Vert +2\int_{0}^{1}\Vert (1-\lambda)x+\lambda y\Vert \,d\lambda } \le&\Vert x\Vert +\Vert y\Vert -\Vert x+y\Vert \\ \le&\frac{2(\Vert x\Vert \Vert y\Vert -\langle x,y\rangle)}{\Vert x+y\Vert +2\int_{0}^{1}\Vert (1-\lambda)x+\lambda y\Vert \,d\lambda}. \end{aligned}$$

### Proof

We have $\Vert x+y\Vert ^{2}=\langle x+y,x+y\rangle=\Vert x\Vert ^{2}+2\langle x,y\rangle+\Vert y\Vert ^{2}$, which implies

$$\Vert x+y\Vert ^{2}=\bigl(\Vert x\Vert +\Vert y\Vert \bigr)^{2}-2\bigl(\Vert x\Vert \Vert y\Vert - \langle x,y\rangle \bigr), $$

which means that

$$\frac{2(\Vert x\Vert \Vert y\Vert - \langle x,y\rangle)}{\Vert x\Vert +\Vert y\Vert +\Vert x+y\Vert }=\Vert x\Vert +\Vert y\Vert -\Vert x+y\Vert . $$

Using point (i) from Theorem 1 in the above equality we obtain the inequalities of the statement. □

### Remark 2

Inequality (3) represents an improvement of the Cauchy-Schwarz inequality.

## Estimates of the triangle inequality using the Tapia semi-product

The Tapia semi-product on the normed space *X* (see [21]) is the function $(\cdot,\cdot)_{T}:X\times X\to\mathbb{R}$, defined by

$$(x,y)_{T}=\lim_{t\searrow0}\frac{\varphi(x+ty)-\varphi(x)}{t}, $$

where $\varphi(x)=\frac{1}{2}\Vert x\Vert ^{2}$, $x\in X$. The above limit exists for any pair of elements $x,y\in X$. The Tapia semi-product is positive homogeneous in each argument and satisfies the inequality $\vert (x,y)_{T}\vert \le \Vert x\Vert \Vert y\Vert $ for all $x,y\in X$. In the case when the norm $\Vert \cdot \Vert $ is generated by an inner product $\langle\cdot,\cdot\rangle$, then $(x,y)_{T}=\langle x,y\rangle$, for all $x,y\in X$.

For instance, in $\mathbb{R}^{n}$, with *p*-norm, $1< p<\infty$, $\Vert x\Vert = (\sum_{i=1}^{n}\vert x_{i}\vert ^{p} )^{1/p}$, for $x=(x_{1},x_{2},\ldots,x_{n})$, the Tapia semi-product becomes

$$(x,y)_{T}=\frac{\sum_{i=1}^{n}\alpha_{i}y_{i}\vert x_{i}\vert ^{p-1}}{ (\sum_{i=1}^{n} \vert x_{i}\vert ^{p} )^{\frac{p-2}{p}}}, $$

if $x\neq0$, where

$$\alpha_{i}=\left \{ \textstyle\begin{array}{l@{\quad}l} 1,&\mbox{if } x_{i}=0,\\ \frac{x_{i}}{\vert x_{i}\vert },&\mbox{otherwise.} \end{array}\displaystyle \right . $$

For nonzero elements $x,y\in X$ denote

4 $$ v(x,y)=\frac{x}{\Vert x\Vert }+\frac{y}{\Vert y\Vert }. $$

First from the Maligranda result we deduce the following inequality.

### Theorem 2

*Let* $x,y\in X$, *be nonzero vectors*. *Then we have*

5 $$ (x,y)_{T}\le \Vert x\Vert \cdot \Vert y\Vert \bigl(\bigl\Vert v(x,y)\bigr\Vert -1\bigr). $$

### Proof

If in the left inequality of Theorem A we replace *y* by *ty*, with $t>0$, $t<\Vert x\Vert /\Vert y\Vert $ and then we divide them by *t* we obtain

$$\bigl(2-\bigl\Vert v(x,y)\bigr\Vert \bigr)\Vert y\Vert \le \Vert y \Vert -\frac{\Vert x+yt\Vert -\Vert x\Vert }{t}. $$

Letting $t\to0$ we obtain

$$\bigl(2-\bigl\Vert v(x,y)\bigr\Vert \bigr)\Vert y\Vert \le \Vert y \Vert -\frac{(x,y)_{T}}{\Vert x\Vert }. $$

From this we obtain immediately equation (5). □

### Remark 3

Inequality (5) is an improvement of the known inequality $(x,y)_{T}\le \Vert x\Vert \cdot \Vert y\Vert $, since $\Vert v(x,y)\Vert \le2$.

### Theorem 3

*Let* $x,y\in X$, *be nonzero vectors such that* $\Vert y\Vert \le \Vert x\Vert $ *and* $\Vert x\Vert y\neq -\Vert y\Vert x$. *Then*

6 $$\begin{aligned}& \Vert x\Vert +\Vert y\Vert -\Vert x+y\Vert \ge \bigl(2-\bigl\Vert v(x,y)\bigr\Vert \bigr)\Vert x\Vert \\& \hphantom{\Vert x\Vert +\Vert y\Vert -\Vert x+y\Vert \ge} {}- \biggl(1- \biggl(\frac{x+y}{\Vert x+y\Vert },\frac {y}{\Vert y\Vert } \biggr)_{T} \biggr) \bigl(\Vert x\Vert -\Vert y\Vert \bigr), \end{aligned}$$

7 $$\begin{aligned}& \Vert x\Vert +\Vert y\Vert -\Vert x+y\Vert \le \bigl(2-\bigl\Vert v(x,y)\bigr\Vert \bigr)\Vert x\Vert \\& \hphantom{\Vert x\Vert +\Vert y\Vert -\Vert x+y\Vert \le}{} - \biggl(1- \biggl(\frac{v(x,y)}{\Vert v(x,y)\Vert },\frac{y}{\Vert y\Vert } \biggr)_{T} \biggr) \bigl(\Vert x\Vert -\Vert y\Vert \bigr), \end{aligned}$$

8 $$\begin{aligned}& \Vert x\Vert +\Vert y\Vert -\Vert x+y\Vert \ge \bigl(2-\bigl\Vert v(x,y)\bigr\Vert \bigr)\Vert y\Vert \\& \hphantom{\Vert x\Vert +\Vert y\Vert -\Vert x+y\Vert \ge}{} + \biggl(1- \biggl(\frac{x+y}{\Vert x+y\Vert },\frac {x}{\Vert x\Vert } \biggr)_{T} \biggr) \bigl(\Vert x\Vert -\Vert y\Vert \bigr), \end{aligned}$$

9 $$\begin{aligned}& \Vert x\Vert +\Vert y\Vert -\Vert x+y\Vert \le \bigl(2-\bigl\Vert v(x,y)\bigr\Vert \bigr)\Vert y\Vert \\& \hphantom{\Vert x\Vert +\Vert y\Vert -\Vert x+y\Vert \le }{} + \biggl(1- \biggl(\frac{v(x,y)}{\Vert v(x,y)\Vert },\frac{x}{\Vert x\Vert } \biggr)_{T} \biggr) \bigl(\Vert x\Vert -\Vert y\Vert \bigr). \end{aligned}$$

### Proof

We choose the following notations:

10 $$ z=\frac{x}{\Vert x\Vert }, \quad\quad u=\frac{y}{\Vert y\Vert }, \quad\quad \lambda= \frac{\Vert y\Vert }{\Vert x\Vert }, \quad\quad \mu=\frac{\Vert x\Vert }{\Vert y\Vert }. $$

For obtaining the first two inequalities we consider the function $f:[0,\infty)\to\mathbb{R}$,

$$f(s)=1+s-\Vert z+s u\Vert , \quad s\in[0,\infty). $$

Also denote

$$g(s)=\Vert z+s u\Vert , \quad s\in[0,\infty). $$

The conditions given in the theorem ensure that $z+su\neq0$, for $s\in [0,\infty)$. We have

$$\begin{aligned} \biggl(\frac{z+s u}{\Vert z+s u\Vert },u \biggr)_{T} =&\frac{1}{\Vert z+s u\Vert }(z+su,u)_{T} \\ =&\lim_{t\searrow0}\frac{\Vert z+(s+t)u\Vert ^{2}-\Vert z+s u\Vert ^{2}}{2t\Vert z+s u\Vert } \\ =&\lim_{t\searrow0}\frac{\Vert z+(s+t)u\Vert -\Vert z+s u\Vert }{t} \\ =&\lim_{t\searrow0}\frac{g(s+t)-g(s)}{t} \\ =&g'(s+0), \end{aligned}$$

where we denoted by $g'(s+0)$ the right derivative of *g* at point *s*. Then

11 $$ f'(s+0)=1- \biggl(\frac{z+s u}{\Vert z+s u\Vert },u \biggr)_{T},\quad s\in[0,\infty). $$

We show that for $\lambda\in[0,1]$ we have

12 $$ f(1)-(1-\lambda)f'(\lambda+0)\le f(\lambda)\le f(1)-(1-\lambda)f'(1+0). $$

Because these inequalities are obvious for $\lambda=1$, we suppose that $0\le\lambda<1$.

Choose an arbitrary number $t>1$. The function *f* is the difference between a linear and a convex function. Then it is concave. Consequently we have

$$\frac{t-1}{t-\lambda}f(\lambda)+\frac{1-\lambda}{t-\lambda }f(t)\le f(1). $$

Straightforward computations shows that this inequality can be written equivalently, in the form

$$f(\lambda)\le f(1)-(1-\lambda)\frac{f(t)-f(1)}{t-1}. $$

If we pass to the limit $t\to1$, $t>1$ in this inequality we obtain the right side inequality in (12).

Also, choose an arbitrary number $\lambda< t<1$. Since *f* is concave we have

$$\frac{t-\lambda}{1-\lambda}f(1)+\frac{1-t}{1-\lambda}f(\lambda )\le f(t). $$

After short computations, this inequality can be written equivalently, in the form

$$f(1)-(1-\lambda)\frac{f(t)-f(\lambda)}{t-\lambda}\le f(\lambda). $$

Passing to the limit $t\to\lambda$, $t>\lambda$ we obtain the left side inequality in (12).

From equation (11) we obtain

13 $$ f'(\lambda+0)=1- \biggl(\frac{x+y}{\Vert x+y\Vert }, \frac {y}{\Vert y\Vert } \biggr)_{T}, \quad\quad f'(1+0)=1- \biggl( \frac {v(x,y)}{\Vert v(x,y)\Vert },\frac{y}{\Vert y\Vert } \biggr)_{T}. $$

If we multiply equations (12) by $\Vert x\Vert $ and we take into account relations (13) and also the following relations:

$$\begin{aligned}& \Vert x\Vert f(\lambda) = \Vert x\Vert +\Vert y\Vert -\Vert x+y \Vert , \\& \Vert x\Vert f(1) = \Vert x\Vert \bigl(2-\bigl\Vert v(x,y)\bigr\Vert \bigr), \\& \Vert x\Vert (1-\lambda) = \Vert x\Vert -\Vert y\Vert , \end{aligned}$$

we deduce inequalities (6) and (7).

In order to obtain the last two inequalities we consider the function $f_{1}:[1,\infty)\to\mathbb{R}$,

$$f_{1}(s)=1+s-\Vert s z+u\Vert , \quad s\in[1,\infty). $$

The right derivatives of the function $f_{1}$ can be obtained immediately from the derivatives of the function *f* by interchanging *u* and *z*, see equation (11). So we obtain

14 $$ f_{1}'(s+0)=1- \biggl(\frac{sz+u}{\Vert sz+u\Vert },z \biggr)_{T},\quad s\in[0,\infty). $$

For $\mu\ge1$ we show the double inequality:

15 $$ f_{1}(1)+f_{1}'(\mu+0) (\mu-1)\le f_{1}(\mu)\le f_{1}(1)+f_{1}'(1+0) ( \mu-1). $$

We consider only the case $\mu>1$, since for $\mu=1$ these inequalities are obvious.

First take an arbitrary number $t>\mu$. Since $f_{1}$ is concave, we have

$$\frac{t-\mu}{t-1}f_{1}(1)+\frac{\mu-1}{t-1}f_{1}(t)\le f_{1}(\mu). $$

This inequality can be rewritten in the equivalent form

$$f_{1}(1)+\frac{f_{1}(t)-f_{1}(\mu)}{t-\mu}(\mu-1)\le f_{1}(\mu). $$

If we pass to the limit $t\to\mu$, $t>\mu$ we obtain the left side inequality in (15).

Next let us take an arbitrary number $1< t<\mu$. Since $f_{1}$ is concave we have

$$\frac{t-1}{\mu-1}f_{1}(\mu)+\frac{\mu-t}{\mu-1}f_{1}(1)\le f_{1}(t). $$

We can transform this inequality to the form

$$f_{1}(\mu)\le f_{1}(1)+\frac{f_{1}(t)-f_{1}(1)}{t-1}(\mu-1). $$

If we pass to the limit $t\to1$, $t>1$ we obtain the right side inequality in (15). If we multiply inequalities given in (15) by $\Vert y\Vert $ and take into account the relations

$$\begin{aligned}& \Vert y\Vert f_{1}(\mu) = \Vert x\Vert +\Vert y\Vert - \Vert x+y\Vert , \\& \Vert y\Vert f_{1}(1) = \Vert y\Vert \bigl(2-\bigl\Vert v(x,y)\bigr\Vert \bigr), \\& f_{1}'(1+0) = 1- \biggl(\frac{v(x,y)}{\Vert v(x,y)\Vert }, \frac{x}{\Vert x\Vert } \biggr)_{T}, \\& f_{1}'(\mu+0) = 1- \biggl(\frac{x+y}{\Vert x+y\Vert }, \frac {x}{\Vert x\Vert } \biggr)_{T}, \\& \Vert y\Vert (\mu-1) = \Vert x\Vert -\Vert y\Vert , \end{aligned}$$

we obtain relations (8) and (9). □

### Remark 4

Inequalities (7) and (8) improve inequality (1).

### Example 2

Let the space *X* and the vectors *x*, *y* be exactly like in Example 1. Then we have $\Vert x\Vert +\Vert y\Vert -\Vert x+y\Vert =\mu(1-\eta )$. Also $v(x,y)=(1,1)+(-1,\eta)=(0,1+\eta)$. Then $(2-\Vert v(x,y)\Vert )\Vert x\Vert =1-\eta$. Hence the right inequality in (1) of Theorem A reads $\mu(1-\eta)\le1-\eta$. We obtain an improvement of this inequality by using inequality (7) given in Theorem 2. Indeed we have

$$\biggl(\frac{v(x,y)}{\Vert v(x,y)\Vert },\frac{y}{\Vert y\Vert } \biggr)_{T}= \bigl((0,1),(-1,\eta) \bigr)_{T} =\lim_{t\searrow0} \frac{1}{2t} \bigl(\bigl\Vert (-t,1+t\eta)\bigr\Vert ^{2}- \bigl\Vert (0,1)\bigr\Vert ^{2} \bigr)=\eta. $$

It follows $(1- (\frac{v(x,y)}{\Vert v(x,y)\Vert },\frac{y}{\Vert y\Vert } )_{T} )(\Vert x\Vert -\Vert y\Vert )=(1-\eta)(1-\mu)$. Consequently inequality (7) from Theorem 2 becomes $\mu(1-\eta)\le\mu(1-\eta)$.

It is easy to see that we can write $\alpha[x,y]=\Vert v(x,-y)\Vert $. Using inequalities (7) and (8) we deduce the following double inequality.

### Corollary 2

*For nonzero vectors* *x* *and* *y*, *such that* $\Vert x\Vert y\neq \Vert y\Vert x$,

16 $$ \frac{\Vert x-y\Vert -\vert \Vert x\Vert -\Vert y\Vert \vert }{\min\{\Vert x\Vert ,\Vert y\Vert \}}+A\le\alpha[x,y]\le\frac {\Vert x-y\Vert +\vert \Vert x\Vert -\Vert y\Vert \vert }{\max\{\Vert x\Vert ,\Vert y\Vert \}}-B, $$

*where*

$$\begin{aligned}& A = \biggl[1- \biggl(\frac{x-y}{\Vert x-y\Vert },\frac {x}{\Vert x\Vert } \biggr)_{T} \biggr]\frac{\vert \Vert x\Vert -\Vert y\Vert \vert }{\min\{\Vert x\Vert ,\Vert y\Vert \}}\ge 0, \\& B = \biggl[1- \biggl(\frac{v(x,-y)}{\Vert v(x,-y)\Vert },-\frac{y}{\Vert y\Vert } \biggr)_{T} \biggr]\frac{\vert \Vert x\Vert -\Vert y\Vert \vert }{\max\{\Vert x\Vert ,\Vert y\Vert \}}\ge 0. \end{aligned}$$

### Proof

Because of the symmetry we can consider that $\Vert x\Vert \ge \Vert y\Vert $. □

### Remark 5

Inequalities (16) improve the inequalities for the norm-angular distance, of Maligranda [4], which can be obtained from (1). Other inequalities for the norm-angular distance could be obtained combining all inequalities (6), (7), (8), and (9).

## Inequalities in inner product spaces

In this section we derive inequalities in an inner product space $(X,\langle\cdot,\cdot\rangle)$ from Theorem 3, by taking into account that $(x,y)_{T}=\langle x,y \rangle$, $x,y\in X$.

### Theorem 4

*Let* $(X,\langle\cdot,\cdot\rangle)$ *be an inner product space*, *with norm* $\Vert \cdot \Vert $. *For nonzero elements* $x,y\in X$

17 $$ \Vert x\Vert +\Vert y\Vert -\Vert x+y\Vert \le \biggl(1-\frac{1}{2} \bigl\Vert v(x,y)\bigr\Vert \biggr) \bigl(\Vert x\Vert +\Vert y\Vert \bigr), $$

*where*

18 $$ \bigl\Vert v(x,y)\bigr\Vert =\biggl\Vert \frac{x}{\Vert x\Vert }+ \frac{y}{\Vert y\Vert }\biggr\Vert =\sqrt{2 \biggl(1+\frac{\langle x,y\rangle}{ \Vert x\Vert \cdot \Vert y\Vert } \biggr)}. $$

### Proof

Because of the symmetry we can suppose that $\Vert x\Vert \ge \Vert y\Vert $. First we have

$$\bigl\Vert v(x,y)\bigr\Vert ^{2}= \biggl\langle \frac{x}{\Vert x\Vert }+ \frac{y}{\Vert y\Vert }, \frac{x}{\Vert x\Vert }+\frac{y}{ \Vert y\Vert } \biggr\rangle =2 \biggl(1+ \frac{\langle x,y\rangle}{ \Vert x\Vert \cdot \Vert y\Vert } \biggr). $$

Hence we get (18). Next we obtain

19 $$\begin{aligned} \biggl(\frac{v(x,y)}{\Vert v(x,y)\Vert },\frac{y}{\Vert y\Vert } \biggr)_{T} =& \frac{1}{\Vert y\Vert \cdot \Vert v(x,y)\Vert } \langle\frac{x}{\Vert x\Vert }+\frac{y}{\Vert y\Vert },y\rangle \\ =&\frac{1}{\Vert v(x,y)\Vert } \biggl(\frac{\langle x,y\rangle}{ \Vert x\Vert \cdot \Vert y\Vert }+1 \biggr) \\ =&\frac{1}{2}\bigl\Vert v(x,y)\bigr\Vert . \end{aligned}$$

We can apply Theorem 3. From equation (7), by taking into account equation (19) we obtain

$$\begin{aligned} \Vert x\Vert +\Vert y\Vert -\Vert x+y\Vert \le& \bigl(2-\bigl\Vert v(x,y)\bigr\Vert \bigr)\Vert x\Vert \\ &{}- \biggl(1- \biggl(\frac{v(x,y)}{\Vert v(x,y)\Vert },\frac{y}{\Vert y\Vert } \biggr)_{T} \biggr) \bigl(\Vert x\Vert -\Vert y\Vert \bigr) \\ =&\bigl(2-\bigl\Vert v(x,y)\bigr\Vert \bigr)\Vert x\Vert - \biggl(1- \frac{1}{2}\bigl\Vert v(x,y)\bigr\Vert \biggr) \bigl(\Vert x\Vert - \Vert y\Vert \bigr) \\ =& \biggl(1-\frac{1}{2} \bigl\Vert v(x,y)\bigr\Vert \biggr) \bigl( \Vert x\Vert +\Vert y\Vert \bigr). \end{aligned}$$

 □

### Remark 6

From the proof we can see that, in an inner product space, inequality (17) is equivalent to inequality (7). In a similar way we can obtain

$$\begin{aligned}& \bigl(2-\bigl\Vert v(x,y)\bigr\Vert \bigr)+ \biggl(1- \biggl\langle \frac {v(x,y)}{\Vert v(x,y)\Vert },\frac{x}{\Vert x\Vert } \biggr\rangle \biggr) \bigl(\Vert x\Vert -\Vert y\Vert \bigr) \\& \quad = \bigl(2-\bigl\Vert v(x,y)\bigr\Vert \bigr)+ \biggl(1-\frac{1}{2} \bigl\Vert v(x,y)\bigr\Vert \biggr) \bigl(\Vert x\Vert -\Vert y\Vert \bigr) \\& \quad = \biggl(1-\frac{1}{2} \bigl\Vert v(x,y)\bigr\Vert \biggr) \bigl( \Vert x\Vert +\Vert y\Vert \bigr). \end{aligned}$$

This means that, if *X* is an inner product space, inequality (9) is also equivalent to inequality (17). Thus in an inner product space equations (7) and (9) are equivalent.

### Remark 7

Inequality (17) can be written equivalently on the form

20 $$ \bigl\Vert v(x,y)\bigr\Vert \le\frac{2\Vert x+y\Vert }{\Vert x\Vert +\Vert y\Vert }, \quad x\neq0, y \neq0. $$

By changing *y* by −*y* in (20) and taking into account that $\alpha[x,y]=\Vert v(x,-y)\Vert $, we see that we have the Dunkl-Williams inequality in an inner product space; see [17, 18].

Inverse inequalities are given in the next theorem.

### Theorem 5

*Let* $(X,\langle\cdot,\cdot\rangle)$ *be an inner product space*, *with norm* $\Vert \cdot \Vert $ *and let nonzero elements* $x,y\in X$ *be such that* $x+y\neq0$.

(i) *If* $\Vert x\Vert \ge \Vert y\Vert $, *then*
  21 $$\begin{aligned}& \Vert x\Vert +\Vert y\Vert -\Vert x+y\Vert \\& \quad\ge \Vert x\Vert +\Vert y\Vert -\bigl\Vert v(x,y)\bigr\Vert \cdot \Vert y\Vert -\frac {\langle x+y,x\rangle(\Vert x\Vert -\Vert y\Vert )}{\Vert x+y\Vert \cdot \Vert x\Vert } \end{aligned}$$
  *and*
  22 $$\begin{aligned}& \Vert x\Vert +\Vert y\Vert -\Vert x+y\Vert \\& \quad\ge \Vert x\Vert +\Vert y\Vert -\bigl\Vert v(x,y)\bigr\Vert \cdot \Vert x\Vert +\frac {\langle x+y,y\rangle(\Vert x\Vert -\Vert y\Vert )}{\Vert x+y\Vert \cdot \Vert y\Vert }. \end{aligned}$$
(ii) *Without condition* $\Vert x\Vert \ge \Vert y\Vert $,
  23 $$\begin{aligned} \Vert x\Vert +\Vert y\Vert -\Vert x+y\Vert \ge& \biggl(1- \frac{1}{2} \bigl\Vert v(x,y)\bigr\Vert \biggr) \bigl(\Vert x\Vert +\Vert y\Vert \bigr) \\ &{}+ \biggl(\frac{1}{4} \bigl\Vert v(x,y)\bigr\Vert ^{2}-1 \biggr)\frac {(\Vert x\Vert -\Vert y\Vert )^{2}}{\Vert x+y\Vert }. \end{aligned}$$

### Proof

(i) We can apply Theorem 3. From equation (8) we obtain

$$\begin{aligned}& \Vert x\Vert +\Vert y\Vert -\Vert x+y\Vert \\& \quad \ge \bigl(2-\bigl\Vert v(x,y)\bigr\Vert \bigr)\Vert y\Vert + \biggl(1- \frac{\langle x,y\rangle+\Vert x\Vert ^{2}}{\Vert x+y\Vert \cdot \Vert x\Vert } \biggr) \bigl(\Vert x\Vert -\Vert y\Vert \bigr) \\& \quad = \Vert x\Vert +\Vert y\Vert -\bigl\Vert v(x,y)\bigr\Vert \cdot \Vert y\Vert -\frac{\langle x+y,x\rangle(\Vert x\Vert -\Vert y\Vert )}{\Vert x+y\Vert \cdot \Vert x\Vert }. \end{aligned}$$

So we proved inequality (21).

We apply Theorem 3. From equation (6) we deduce that

$$\begin{aligned}& \Vert x\Vert +\Vert y\Vert -\Vert x+y\Vert \\& \quad \ge \bigl(2-\bigl\Vert v(x,y)\bigr\Vert \bigr)\Vert x\Vert - \biggl(1- \frac{\langle x,y\rangle+\Vert y\Vert ^{2}}{\Vert x+y\Vert \cdot \Vert y\Vert } \biggr) \bigl(\Vert x\Vert -\Vert y\Vert \bigr) \\& \quad = \Vert x\Vert +\Vert y\Vert -\bigl\Vert v(x,y)\bigr\Vert \cdot \Vert x\Vert +\frac{\langle x+y,y\rangle(\Vert x\Vert -\Vert y\Vert )}{\Vert x+y\Vert \cdot \Vert y\Vert }. \end{aligned}$$

So we proved inequality (22) too.

(ii) By virtue of the symmetry of equation (23) we can suppose that $\Vert x\Vert \ge \Vert y\Vert $. If we add inequalities (21) and (22) and then we divide by 2 we obtain

$$\begin{aligned}& \Vert x\Vert +\Vert y\Vert -\Vert x+y\Vert \\& \quad \ge \Vert x\Vert + \Vert y\Vert -\frac{1}{2} \bigl\Vert v(x,y)\bigr\Vert \bigl(\Vert x\Vert +\Vert y\Vert \bigr) \\& \quad\quad{} +\frac{\langle x,y\rangle(\Vert x\Vert -\Vert y\Vert )^{2}}{2\Vert x\Vert \cdot \Vert y\Vert \cdot \Vert x+y\Vert }-\frac{(\Vert x\Vert -\Vert y\Vert )^{2}}{2\Vert x+y\Vert } \\& \quad = \biggl(1-\frac{1}{2} \bigl\Vert v(x,y)\bigr\Vert \biggr) \bigl(\Vert x\Vert +\Vert y\Vert \bigr)+ \biggl(\frac{1}{4} \bigl\Vert v(x,y)\bigr\Vert ^{2}-1 \biggr)\frac{(\Vert x\Vert -\Vert y\Vert )^{2}}{\Vert x+y\Vert }. \end{aligned}$$

 □

### Remark 8

From the proof of Theorem 5 it follows that in an inner product space equation (21) is equivalent to equation (8) and equation (22) is equivalent to equation (6).

### Remark 9

Inequality (23) has the advantage of the symmetry, but in general it does not improve the triangle inequality.

### Remark 10

We note also that inequality (21) is equivalent to inequality (20). Indeed, equation (21) can be written in equivalent form,

$$\Vert x\Vert \cdot \Vert x+y\Vert ^{2}\le \bigl(\Vert x \Vert ^{2} +\langle x,y\rangle\bigr) \bigl(\Vert x\Vert -\Vert y \Vert \bigr)+\bigl\Vert v(x,y)\bigr\Vert \cdot \Vert x\Vert \cdot \Vert y\Vert \cdot \Vert x+y\Vert . $$

Then

$$\bigl(\Vert x\Vert +\Vert y\Vert \bigr) \biggl(\frac{\langle x,y\rangle }{\Vert x\Vert +\Vert y\Vert }+1 \biggr)\le \bigl\Vert v(x,y)\bigr\Vert \cdot \Vert x+y\Vert , $$

and then

$$\bigl\Vert v(x,y)\bigr\Vert ^{2}\le\bigl\Vert v(x,y)\bigr\Vert \cdot\frac{2\Vert x+y\Vert }{\Vert x\Vert +\Vert y\Vert }. $$

If $v(x,y)\neq0$ we obtain (20). If $v(x,y)=0$ then equation (20) is obvious but also equation (21) is obvious, since $v(x,y)=0$ implies $\Vert x\Vert y=-\Vert y\Vert x$ and then $\Vert x+y\Vert =\Vert x\Vert -\Vert y\Vert $, then inequality (21) is reduced to an equality.

Also inequality (22) is equivalent to inequality (20). Indeed, equation (22) can be written in equivalent form,

$$\bigl(\Vert y\Vert ^{2} +\langle x,y\rangle\bigr) \bigl(\Vert x \Vert -\Vert y\Vert \bigr)+\Vert y\Vert \cdot \Vert x+y\Vert ^{2}\le\bigl\Vert v(x,y)\bigr\Vert \cdot \Vert x\Vert \cdot \Vert y\Vert \cdot \Vert x+y\Vert , $$

and then

$$\bigl\Vert v(x,y)\bigr\Vert ^{2}\le\bigl\Vert v(x,y)\bigr\Vert \cdot\frac{2\Vert x+y\Vert }{\Vert x\Vert +\Vert y\Vert }. $$

From this, we reason similarly to in the case of equation (21).

In conclusion, all relations (17), (20), (21), (22), and consequently also (23), are equivalent to each other, for $x\neq0$, $y\neq0$, $x+y\neq0$. From Remarks 6 and 8 it follows that if *X* is an inner product space inequalities (6), (7), (8), and (9) are equivalent to each other, for $\Vert x\Vert \ge \Vert y\Vert >0$ and $\Vert x\Vert y\neq-\Vert y\Vert x$.
